# Supplementary figures and images for: High-performance enrichment-based genome sequencing to support the investigation of hepatitis A virus outbreaks
Source: Microbiol Spectr. 2023 Nov 29;12(1):e02834-23. doi: 10.1128/spectrum.02834-23 (PMC10783085; doi:10.1128/spectrum.02834-23)

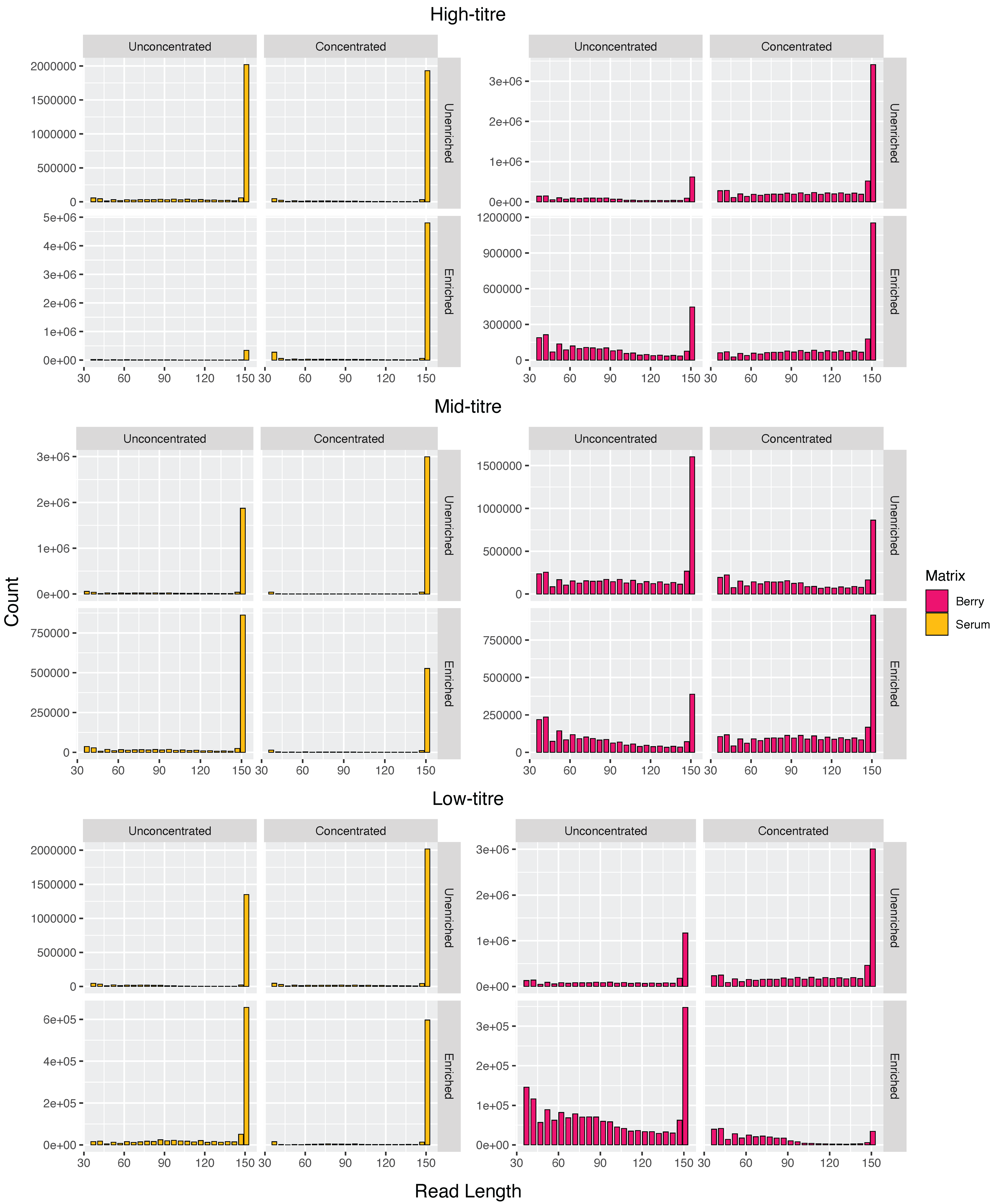

Supplement: Fig. S1 — Read length distributions. [file spectrum.02834-23-s0001.tiff]
